# Hemangioblastoma with late leptomeningeal metastasis: a case report

**DOI:** 10.1186/s13256-023-03812-5

**Published:** 2023-03-20

**Authors:** Spencer J. Poiset, Aneesh Reddy, Catherine M. Tucker, Lawrence C. Kenyon, Kevin D. Judy, Wenyin Shi

**Affiliations:** 1 Department of Radiation Oncology, Sidney Kimmel Medical College, Thomas Jefferson University, 111 S 11 ST, Suite G301, Philadelphia, PA 19107 USA; 2 The College of New Jersey, Ewing, NJ USA; 3 Department of Pathology, Anatomy and Cell Biology, Thomas Jefferson University, Philadelphia, PA USA; 4 Department of Neurological Surgery, Thomas Jefferson University, Philadelphia, PA USA

**Keywords:** Hemangioblastoma, Leptomeningeal disease, Metastasis, Radiation

## Abstract

**Background:**

Hemangioblastoma of the central nervous system is an uncommon benign neoplasm, with about 25% of cases in patients with von Hippel–Lindau disease. The incidence of metastasis is rare, particularly in patients without von Hippel–Lindau disease. We report a case of hemangioblastoma with leptomeningeal dissemination as a late recurrence.

**Case presentation:**

A 65-year-old Caucasian man with a history of World Health Organization grade I hemangioblastoma of the cerebellar vermis underwent gross total resection in 1997. In early 2018, he developed intracranial recurrences with diffuse leptomeningeal disease of the entire spine. The patient underwent resection of intracranial recurrence, followed by palliative craniospinal irradiation. The disease progressed quickly, and he died 8 months after recurrence.

**Conclusions:**

Despite a benign pathology, hemangioblastoma has a low risk of metastasis. The outcome for hemangioblastoma patients with metastasis is poor. Multidisciplinary care for patients with metastatic hemangioblastoma warrants further investigation, and an effective systemic option is urgently needed. Regular lifelong follow-up of at-risk patients is recommended.

## Background

Hemangioblastoma (HB) of the central nervous system is an uncommon benign neoplasm accounting for approximately 2% of intracranial neoplasms and 2–10% of primary spinal cord neoplasms [1]. Primary lesions most commonly arise in the cerebellum, with HBs comprising 7–12% of posterior fossa lesions in adults. While the majority of hemangioblastomas arise sporadically, about 25% of cases are in patients with von Hippel–Lindau (VHL) disease [2]. The presence of multiple lesions should raise suspicions of VHL. Due to the low metastatic potential of HB, disseminated hemangioblastomatosis in patients without VHL is a rare occurrence [2, 3]. We report a case of hemangioblastoma with leptomeningeal dissemination as a late recurrence. This is a very unusual presentation for this benign tumor, and it raised the need for long-term follow-up for patients with hemangioblastoma, particularly in high-risk patients.

## Case presentation

**Fig. 1:**
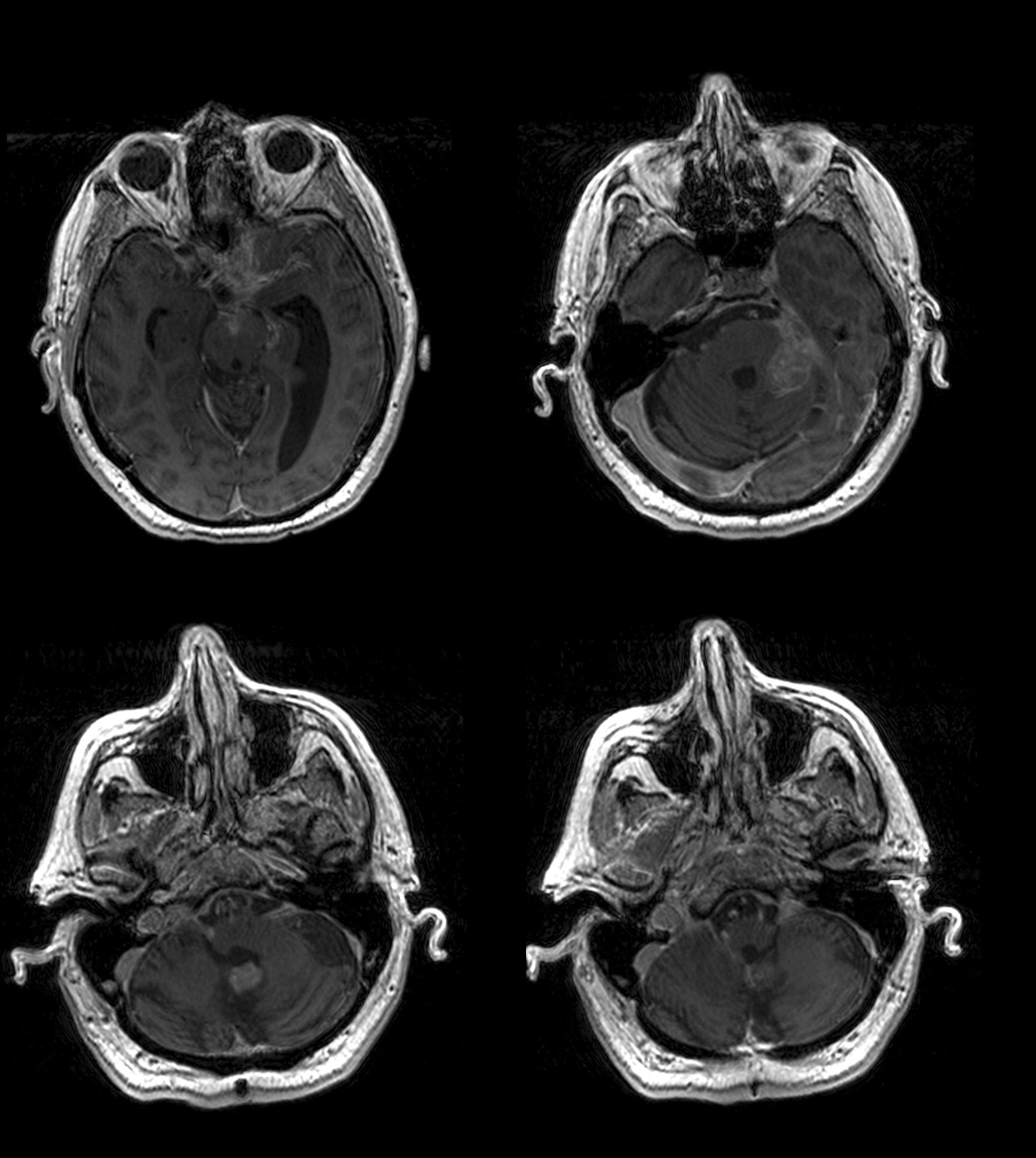
Encephalomalacia in bilateral medial cerebellum. Enhancing soft tissue nodule along anteromedial left cerebellum/vermis and enhancing mass along left cerebellopontine angle. Abnormal enhancement along pre-chiasmatic optic nerves and chiasm, without intraorbital involvement. Leptomeningeal enhancement involving bilateral frontal lobes and bilateral ambient and suprasellar cistern, with extension into interhemispheric fissure

**Fig. 2:**
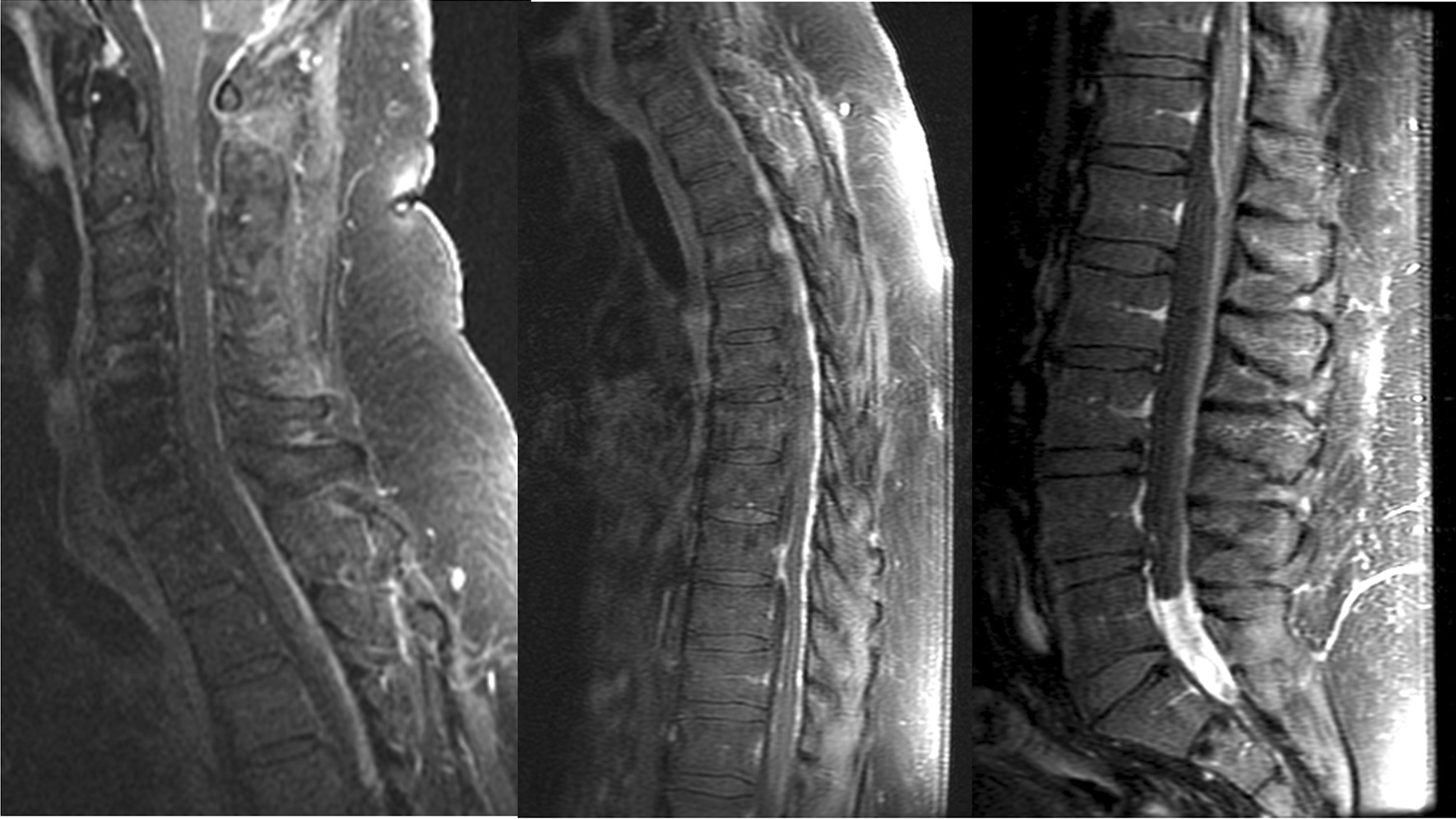
Extensive nodular and linear enhancement in posterior fossa and along the surface of the spinal cord representing leptomeningeal metastasis. Diffuse abnormal plaque-like and nodular enhancement along the cord surface throughout the thoracic spine and clearly identifiable ventral intrathecal tumor below T9. Thick plaque-like enhancing soft tissue within the thecal sac at the L5–S1 level reflecting drop metastasis

A 65-year-old Caucasian man with no significant past medical history presented with a World Health Organization (WHO) grade I hemangioblastoma of the cerebellar vermis post suboccipital craniotomy and resection in 1997. A VHL gene mutation test was not performed then. He had a gross total resection and was observed, and was later lost to follow-up. He presented again in early 2018 with 3 months of forgetfulness, worsening gait imbalance, and ataxia. Physical examination was significant for bilateral upper and lower extremity ataxia, worse in the left lower extremity. Magnetic resonance imaging (MRI) of the brain revealed a 2.8 cm brightly enhancing complex extra-axial solid/cystic mass arising from inferior surface of left tentorium, producing a local mass effect on the superior surface of the left cerebellar hemisphere and a second well-defined, 1 cm brightly enhancing extra-axial mass at the cerebellar vermis. There was also arachnoidal spreading of the tumor, most prominently in the suprasellar cistern and along the sylvian fissures, consistent with diffuse leptomeningeal disease (Fig. 1). Subsequently, a cervical, thoracic, and lumbar spine MRI was ordered, which revealed extensive extramedullary intradural enhancement consistent with extensive leptomeningeal involvement along the entire spinal cord, T8 to T11 cord enhancement, and thick plaque-like enhancing soft tissue within the thecal sac reflecting drop metastases (Fig. 2). A computed tomography (CT) scan of the chest, abdomen and pelvis was negative for metastatic disease. A lumbar puncture was performed, which was nondiagnostic but showed an atypical cerebrospinal fluid (CSF) cytology.

**Fig. 3:**
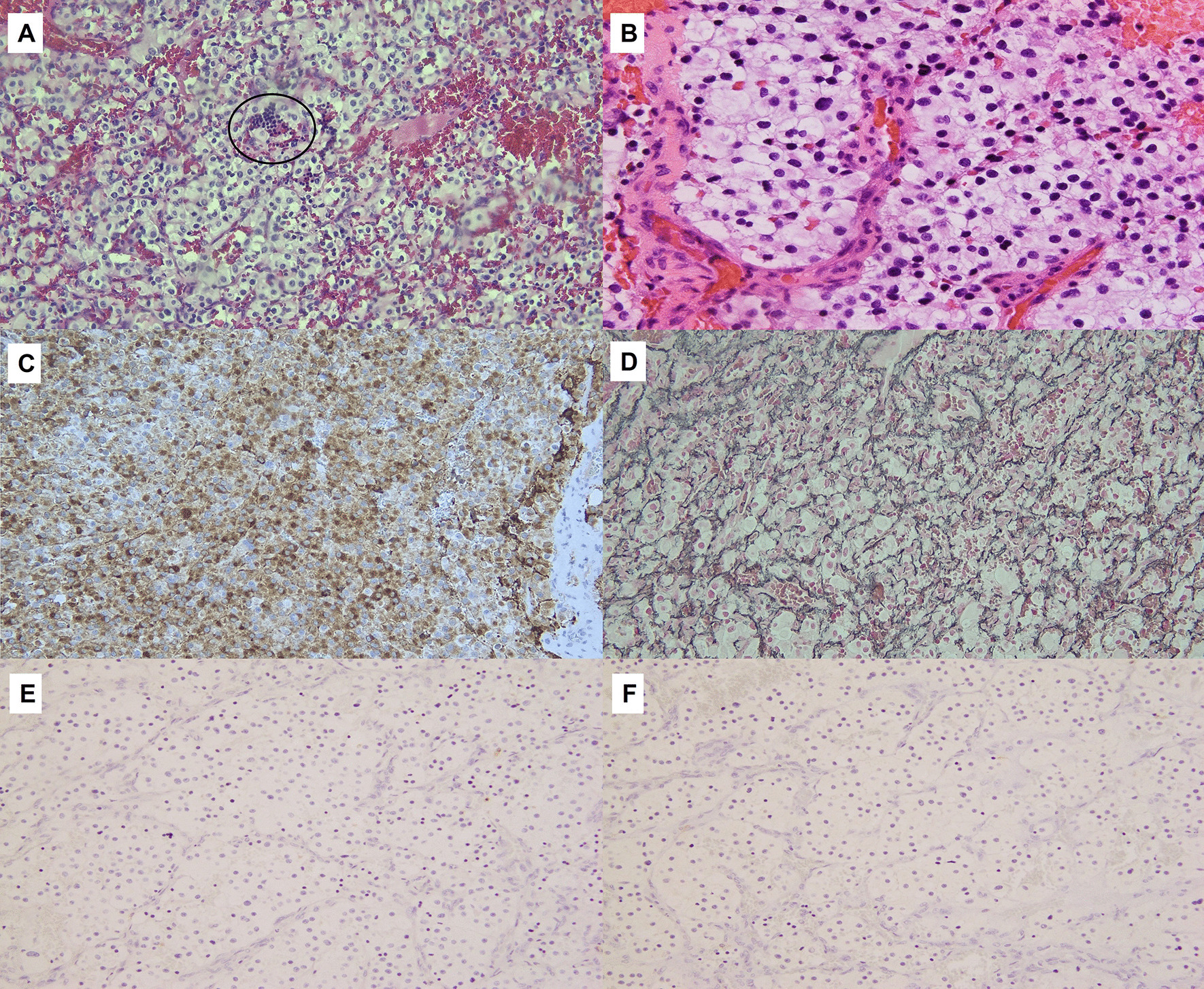
**A**–**F** Lateral cerebellar tumor, histologically consistent with hemangioblastoma (**A**, hematoxylin and eosin stain, 200× magnification). Extramedullary hematopoiesis is seen (**A**, circle). A high-power image shows hemangioblastoma stromal cells with vacuolated cytoplasm surrounded by thin-walled capillaries ( **B**, hematoxylin and eosin stain, 400× magnification). Immunohistochemical staining for inhibin is strongly positive in tumor cells (**C**, 200× magnification). Reticulin stain highlights the delicate capillary network that surrounds the nests of hemangioblastoma stromal cells (**D**, 200× magnification). Cytokeratin CAM 5.2 (**E**, 200× magnification) and renal cell carcinoma (RCC, **F**, 200× magnification) stains are negative in the hemangioblastoma cells, ruling out metastatic renal cell carcinoma

The patient underwent a suboccipital craniotomy for resection of the recurring tumor in the vermis, as well as the new infratentorial tumor. Immunohistochemistry for inhibin was strongly positive within the tumor cells, which supported the diagnosis of hemangioblastoma (Fig. 3). The postoperative course was complicated by communicating hydrocephalus requiring a ventriculoperitoneal shunt (VPS) placement. Subsequently, the patient received palliative craniospinal irradiation. He received 30 Gy in ten fractions to the whole brain and whole spine. The patient tolerated radiation treatment well without significant side effects. MRI of the brain, cervical, thoracic and lumbar spine 2 months post-radiation demonstrated new bilateral complex subdural hygromas, stable diffuse pachymeningeal, and leptomeningeal disease. Approximately 4.5 months post-radiation treatment, the patient presented once again with altered mental state and MRI of the brain revealed increased subdural hygromas, which was worse on the left. He underwent a left frontal craniotomy for evacuation of the left hygroma but his condition deteriorated with admission complicated by aspiration pneumonia, urinary tract infection (UTI), multifactorial encephalitis, and respiratory distress requiring intubation. The patient was placed on comfort care and died from respiratory failure shortly after, 6 months post-radiation and 8 months following presentation with disseminated disease.

## Discussion

Primary hemangioblastomas are rare tumors of the central nervous, often slow growing and most commonly occurring in the cerebellum, brainstem, or spinal cord. Hemangioblastomas can arise sporadically; however, they are classically associated with von Hippel–Lindau (VHL) disease, with approximately 25% of hemangioblastomas being attributed to the disease [1]. Multiple hemangioblastomas can be seen in at least 60% of VHL patients, and is one of the most common causes of demise in VHL patients, in addition to renal cell carcinoma. However, disseminated leptomeningeal involvement of hemangioblastomas is exceedingly rare in patients without a diagnosis of VHL disease, with only ~ 33 published cases [2, 3].

Radiation therapy, either stereotactic radiosurgery or external beam radiotherapy, can be used as primary, adjuvant or a salvage treatment strategy for localized disease with excellent local control [4–6] [16,18,19]. Local control is 98%, 88%, and 73% of intracranial hemangioblastomas at 1, 2, and 6 years, respectively, with marginal dose and fractions ranging from 10–32 Gy and 1–10 fractions, respectively [7]. However, in patients with diffuse leptomeningeal involvement treated with palliative radiation, outcomes are poor [8–10]. In reviewing all prior cases of diffuse leptomeningeal HB, a majority of patients had experienced disease progression or complications due to disease burden, with 79%, 47%, and 18% survival at 1, 2, and 5 years,respectively, in case reports with long-term follow-up. In addition to surgery and radiation, several other systemic therapies have been explored with disappointing results. Systemic therapies also have poor results in patients with metastatic disease [11, 12]. With all reported cases of diffuse leptomeningeal HB, there are similar rates of overall survival in patients with 76%, 45%, and 18% survival at 1, 2, and 5 years, respectively, in patients with long-term follow-up. Our patient died shortly after palliative craniospinal radiation, with progression of disease and subsequent respiratory failure 6 months post-treatment. The clinical course of our patient closely aligns with prior case reports, with a quick declining following palliative radiation in patients with diffuse dissemination of HB in the brain and spine [13–15]. In reported cases, dissemination occurred approximately 8.5 years after the primary hemangioblastoma. As a result, long-term follow-up of these tumors, particularly in multifocal and recurrent patients, may be necessary.

Systemic therapy has also been used for management of metastatic hemangioblastoma. Although anthracycline-based therapies are frequently used, limited data is available regarding its efficacy. Given the rich vascular characteristics of hemangioblastoma, antiangiogenic therapies have also been investigated, such as bevacizumab, imatinib, sorafenib, sunitinib, and pazopanib. However, further studies are needed to better define the optimal systemic therapeutic regimen.

## Conclusion

We report a case of cerebral hemangioblastoma with leptomeningeal metastasis as a late recurrence. Treatment outcomes of hemangioblastoma patients with metastasis are poor. A multidisciplinary care for patients with metastatic hemangioblastoma warrants further investigation and an effective systemic option is urgently need. Regular lifelong follow-up in at-risk (multifocal and recurrent) patients is recommended.

## Data Availability

Data available on request from the authors.
